# p.Asp58Val Hereditary Transthyretin Amyloidosis: A Case Report and Literature Review

**DOI:** 10.7759/cureus.99872

**Published:** 2025-12-22

**Authors:** Jaineel Ramnarain, Patrick Hosking, Simon Gibbs

**Affiliations:** 1 Haematology, Epworth HealthCare, Melbourne, AUS; 2 Haematology, Eastern Health, Melbourne, AUS

**Keywords:** cardiac amyloid, genotype-phenotype association, hereditary transthyretin amyloidosis, imaging modalities, recurrent syncope

## Abstract

Hereditary transthyretin amyloidosis (ATTRv) is a clinically important yet under-recognised entity. It develops when inherited genetic mutations synthesise dysfunctional transthyretin protein, which accrues and disrupts organs, typically causing cardiac failure or polyneuropathy. Whilst numerous genotypes have been implicated, this report aims to provide a detailed account of the rare and pathognomonic aspartate to valine amino acid substitution, p.Asp58Val, affecting a 54-year-old Australian female, along with associated clinical manifestations. Genotypic-phenotypic relationships are also examined within index and prior cases. Of note, recurrent syncope formed a predominant symptom affecting our patient, preceding eventual diagnosis, followed by multidisciplinary management to optimise her level of functioning. In summary, progressive neuropathy followed by cardiomyopathy may represent key clinical features associated with p.Asp58Val hereditary transthyretin amyloidosis. Physician vigilance for these symptoms, especially amongst patients with familial cardiomyopathy, may improve detection of this rare disease.

## Introduction

Amyloidosis describes a heterogeneous group of disorders collectively characterised by abnormal protein folding, aggregation into amyloid fibrils, and extracellular accumulation often culminating in end-organ dysfunction. Systemic amyloidosis predominantly involves disordered light chain immunoglobulin, transthyretin or serum amyloid A [1,2]. On a molecular level, transthyretin represents the key transporter protein of thyroid hormone and retinol, although it may become amyloidogenic via inherited or acquired means. Inheritance of a mutated transthyretin gene coding for a ‘variant’ and unstable transthyretin protein underpins hereditary transthyretin amyloidosis (ATTRv), whereas accelerated degradation of the genotypically normal, i.e., ‘wild-type’ protein in response to ageing describes ‘wild-type’ transthyretin amyloidosis (ATTRwt) [3]. Both forms of transthyretin amyloidosis (ATTR) have a cardiac predilection, with current literature suggesting ATTRv is likely quite prevalent yet under-recognised [4]. The epidemiology of ATTRv accordingly remains unclear despite more than 130 pathogenic mutations and familial clusters identified across the United States, Brazil, Northern Ireland, Portugal, Italy, Poland, Sweden, Korea and Japan [5,6]. Amongst these genetic variants, a rare pathognomonic aspartate to valine amino acid substitution (p.Asp58Val) derived from a missense point mutation has re-emerged [7], with this report detailing only the eighth documented and first Australian case.

## Case presentation

A high-functioning 54-year-old female presented to her local medical officer for evaluation of recurrent falls in October 2021. History revealed several cardiac-related symptoms comprising paroxysmal palpitations, posture-related dissociation, orthostatic pre-syncope and an episode of perioperative syncope over the last several years. A distant history of irritable bowel syndrome and ascending paraesthesia involving her lower limbs in conjunction with mild peripheral oedema was also noted.

Other past medical history included allergic rhinitis, asthma, oesophageal spasm, gastroesophageal reflux, cholelithiasis, hypotension, hypercholesterolaemia, lumbar degeneration and undifferentiated polyarticular arthritis unresponsive to disease-modifying anti-rheumatic drugs. Previous surgeries included a breast reduction, cholecystectomy, lumbar laminectomy, discectomy, spinal fusion and instrumentation, along with the recent insertion of an implantable cardioverter-defibrillator (ICD) as primary prevention in the setting of recurrent syncope: her pre-implantation echocardiogram was reportedly unremarkable. Regular medications involved magnesium, ranitidine and vitamin D, and other than opioid-related nausea, there were no drug allergies. She was a distant ex-smoker of 10 pack-years and did not consume alcohol or partake in any recreational substances.

Family history was significant for hypertrophic cardiomyopathy in her father, who suffered a fatal myocardial infarction when 69 years old. Her mother and elder sister both suffered non-fatal myocardial infarctions, and her mother also reported low back pain. In contrast, both her teenage children remained asymptomatic. Her maternal half-brother also suffered a fatal myocardial infarction at the age of 63 years.

**Figure 1:**
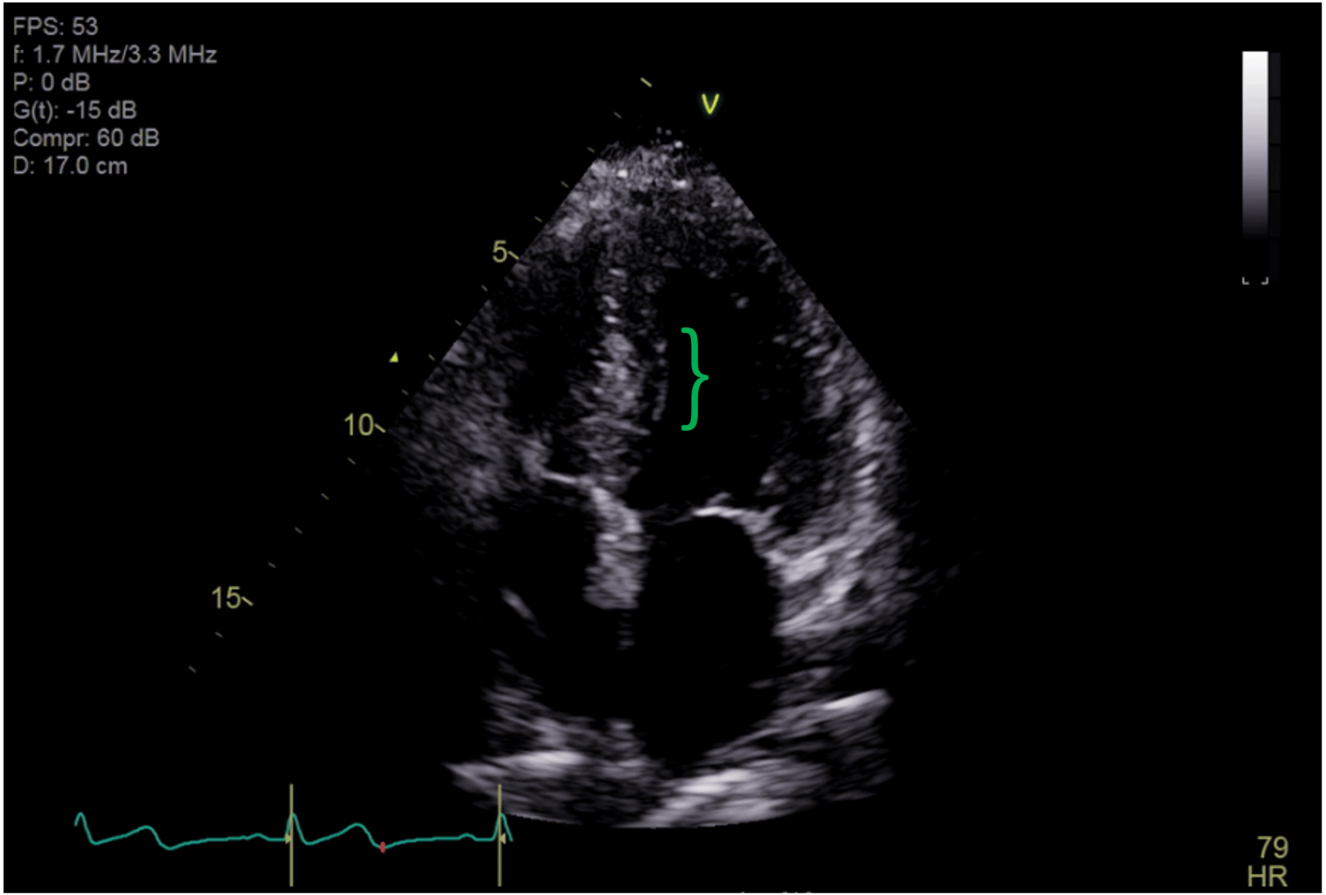
Transthoracic Echocardiogram Transthoracic echocardiogram (TTE) with left ventricular hypertrophy and speckled intraventricular hypertrophy suggestive of cardiac amyloidosis (bracket) [8].

**Figure 2:**
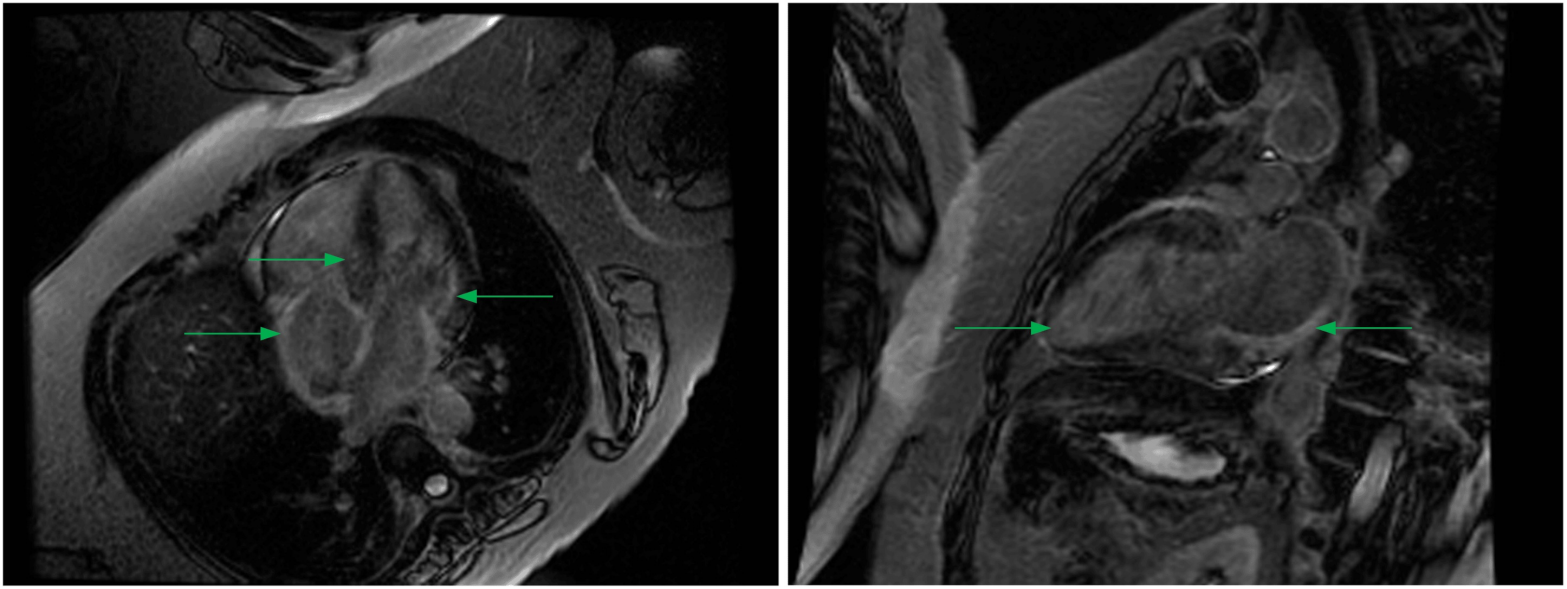
Cardiac Magnetic Resonance Late gadolinium enhancement involving both atria and ventricles on cardiac magnetic resonance (CMR) imaging (arrows).

To differentiate her symptoms, our patient received comprehensive multidisciplinary assessments. After exclusion of neurogenic syncope, subsequent cardiac evaluation found no postural-related hypotension nor tachycardia, although echocardiography unexpectedly revealed concentric left ventricular hypertrophy with low-normal global longitudinal strain measuring 18% without overt apical sparing (Figure 1). Follow-up cardiac magnetic resonance then demonstrated basal inferior and atrial wall late gadolinium enhancement, raising the possibility of amyloidosis as depicted in Figure 2.

**Figure 3:**
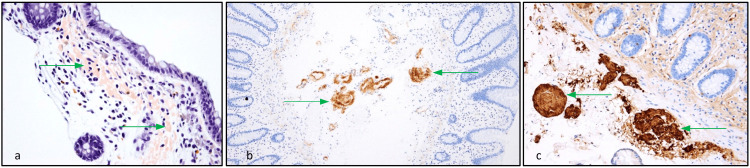
Colonic Histopathology Immunohistochemistry of colonic biopsy samples with (a) Congo-red, (b) amyloid-P 213 protein and (c) transthyretin staining (arrows).

She was accordingly referred to our centre for evaluation. Archived colonoscopy biopsies were retrieved and stained positive for Congo red, which exhibited apple green birefringence under polarising light: the histologic hallmark of amyloid protein. Immunohistochemistry demonstrated kappa, lambda and amyloid-A negativity, whilst amyloid-P stained positive and transthyretin was strongly positive within blood vessel walls, thereby confirming systemic amyloidosis as illustrated in Figure 3. Subsequent genotyping of the transthyretin gene heralded ATTRv with a rare genetic variant, namely an aspartate to valine amino acid substitution arising from a point mutation on chromosome 18q12.1 [7].

**Figure 4:**
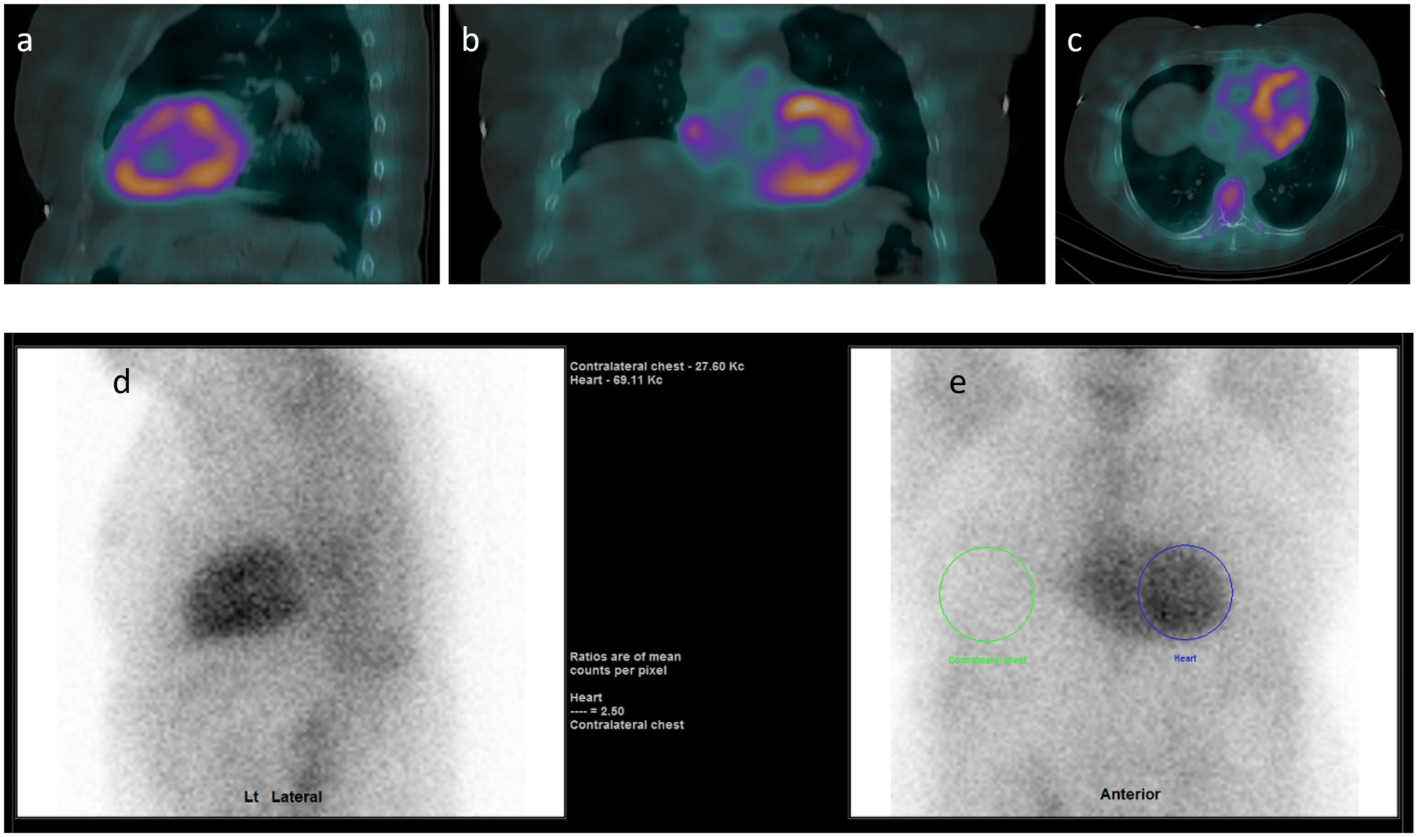
Nuclear Cardiac Scintigraphy Tc-99m pyrophosphate cardiac scintigraphy (PYP) showing coloured left ventricular myocardial avidity (a-d) with cardiothoracic standardised uptake value ratio >1.5 (e) consistent with cardiac amyloidosis [10]. *Tc-99m: Technetium-99m.

Further clinical, biochemical and radiologic investigations with nuclear cardiac scintigraphy (Figure 4) established Gilmore stage I ATTRv [9], given an N-terminal prohormone brain natriuretic peptide (NT-proBNP) of 284 ng/L and an estimated glomerular filtration rate of 88 mL/min/1.73m^2^. There was no plasma cell dyscrasia (kappa free light chains 12.5 mg/L, lambda free light chains 14.5 mg/L, K:L ratio 0.86, paraprotein undetectable), and her preliminary cardiac troponin T measured 33 ng/L in the setting of normal sinus rhythm with a left anterior fascicular block on electrocardiogram. Occasional ventricular ectopic beats and non-sustained ventricular tachycardia were evident on subsequent Holter monitoring without atrial fibrillation or ischaemia. Electromyography (EMG) demonstrated axonal demyelination, confirming peripheral neuropathy, possibly contributing to her falls, aside from apparent autonomic dysfunction.

As such, our patient was commenced on epigallocatechin-3-gallate (EGCG), doxycycline and diflunisal to help mitigate progressive amyloidosis [6]. Both doxycycline and diflunisal were well tolerated, in contrast to EGCG, which was ceased after a few weeks due to excessive diaphoresis. A 1.5 L per day fluid restriction in addition to furosemide and spironolactone was helpful in controlling progressive cardiac failure, titrated to volume status and NT-proBNP. Both pregabalin and amitriptyline were also instituted to address the onset of painful lower limb peripheral neuropathy with good effect [11].

**Figure 5:**
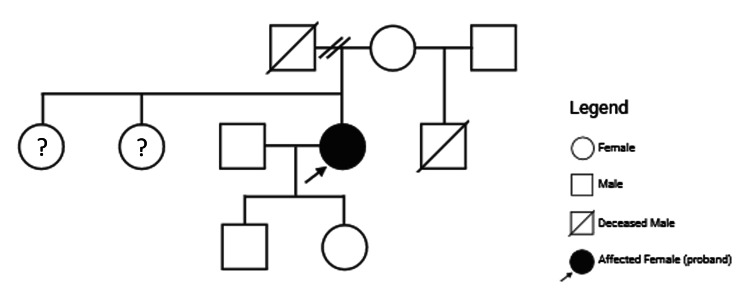
Pedigree Family pedigree including individuals awaiting genetic testing (?).

Given autosomal dominant inheritance, genetic screening of our patient’s relatives was also commenced to identify other individuals at risk, besides garnering further genetic and clinical information to compile a pedigree (Figure 5). Her children were, however, not genotyped as per her wishes.

After 12 months, our patient reported an improvement in postural symptoms, tendency to fall, functional status and overall quality of life, albeit without returning to her premorbid baseline activity. She continues with a close multidisciplinary follow-up to optimise her cardiomyopathy and neuropathic symptoms and functioning.

## Discussion

Amyloidosis describes a spectrum of disorders typified by abnormal amyloid fibril deposition within multiple organs. Despite multisystem involvement, progressive non-specific symptoms often manifest, presenting diagnostic challenges to physicians.

ATTRv is an important clinical entity gaining increasing recognition in recent years, particularly with advancements and availability in genetic testing, for example, next-generation sequencing. Over 130 pathogenic mutations have been identified to date, including a missense point mutation on chromosome 18q12.1, inducing an aspartate to valine amino acid substitution, namely p.Asp58Val. Similar to other mutations such as p.Val142Ile and p.Val50Met [12,13], p.Asp58Val also results in abnormal transthyretin synthesis, misfolding, aggregation and fibril formation, which resists degradation, causing tissue injury [14].

Despite these genetic insights, the clinical phenotype of p.Asp58Val still remains variable. Of the seven previously reported cases [15-19], all affected individuals exhibited varying degrees of neurologic and cardiac sequelae. To illustrate, in 2002, Lachmann and colleagues described a 58-year-old male of Ghanaian ancestry referred to the National Amyloidosis Centre of the United Kingdom [15]. This patient suffered predominant neuropathy with cardiac and splenic amyloid also detected.

Augustin et al. then reported three members of a large Spanish kindred with the same aspartate to valine amino acid substitution denoted D38V (p.Asp58Val) [7,16]. Herein, the proband involved a 71-year-old male exhibiting sensory-motor polyneuropathy with progressive autonomic symptoms encompassing orthostatic hypotension and syncope. He later developed gastrointestinal dysfunction with diarrhoea and weight loss. Severe cardiomyopathy then ensued, culminating in death from acute pulmonary oedema approximately 12 months after his diagnostic TTE. An affected sister presented with mild to moderate cardiomyopathy at age 66, followed by autonomic and gastrointestinal symptoms comparable to the proband. Cardiac amyloidosis was also evident on both TTE and Tc-99m nuclear scintigraphy. In contrast, the proband’s 39-year-old niece with ATTRv remained asymptomatic with normal EMG and TTE.

Almost a decade later, Gillmore and associates discovered a 42-year-old female of African descent within an international multicentre study evaluating suspected or histologically proven amyloidosis; however, no clinical details were provided [17].

In 2020, Kim and colleagues reported a 47-year-old Korean male with the p.Asp58Val mutation [18]. Herein, the authors demonstrated cardiac amyloidosis on TTE, CMR and Tc-99m nuclear scintigraphy along with peripheral nerve and gastrointestinal involvement. Later the same year, Lipowska et al. published another genotypically identical ATTRv case involving a 51-year-old Polish male attending the Outpatient Neuromuscular Clinic of the Medical University of Warsaw [19]. Their patient demonstrated prominent sensorimotor polyneuropathy associated with muscle wasting necessitating a gait aid; autonomic symptoms were also present, including diarrhoea and genitourinary dysfunction. He subsequently developed mild cardiomyopathy with concentric left ventricular hypertrophy evident on both TTE and CMR. Importantly, he described no significant family history. 

Similar to these reports, our patient suffered predominant neurologic and cardiac dysfunction, with her earliest symptoms emerging in her mid-late 40s. More specifically, this entailed autonomic and peripheral neuropathy encompassing gastrointestinal dysfunction and ascending paraesthesia, before cardiomyopathy associated with syncope and fluid overload. As such, progressive neuropathy and cardiomyopathy may be characteristic clinical features of ATTRv involving the p.Asp58Val mutation. More studies are nonetheless required to consolidate these potentially heralding genotypic-phenotypic relationships.

## Conclusions

In summary, we suggest considering ATTRv, especially amongst relatively young patients with undifferentiated cardiomyopathy and neurological phenomena. Subsequent genotyping of suspected individuals may also help to confirm diagnosis and enhance detection rates amidst phenotypic variability. This could, in turn, facilitate access to treatments such as tafamidis, patisiran, monoclonal antibodies, and clustered regularly interspaced short palindromic repeats (CRISPR), which offer potential to significantly alter the natural history of the disease.
